# Negative control of *Candida albicans* biofilm formation by combined action of white-opaque regulator Wor2 and biofilm regulator Bcr1

**DOI:** 10.1093/g3journal/jkag133

**Published:** 2026-05-16

**Authors:** Katharina Goerlich, Aaron P Mitchell

**Affiliations:** Department of Microbiology, University of Georgia, Athens, GA 30602, United States; Department of Microbiology, University of Georgia, Athens, GA 30602, United States

**Keywords:** *Candida albicans*, biofilm formation, filamentation, transcriptional regulation

## Abstract

Biofilm formation is vital for the survival and pathogenicity of the fungus *Candida albicans.* Expression of biofilm-promoting genes is coordinated by a transcription factor network that governs the yeast-filament transition and other processes. A second cell type transition, the white-opaque transition, is coordinated by its own transcription factor network. Initial studies suggested that the 2 transcriptional networks have a mutually exclusive relationship, driven in part by reciprocal repression of biofilm regulator Efg1 and white-opaque regulator Wor1. However, recent studies have shown that biofilm regulators and white-opaque regulators can promote one another's function in many situations. Here, we test the function of white-opaque regulator Wor2 in biofilm formation. We find that Wor2 has a functional relationship with biofilm regulator Bcr1. We characterized the phenotype of *bcr1*Δ/Δ *wor2*Δ/Δ mutants in 5 strain backgrounds and conducted RNA-sequencing (RNA-seq) analysis in the SC5314 reference strain background. The combined Bcr1-Wor2 impact is unexpected: although Bcr1 is known as a positive regulator of biofilm formation and biofilm-related genes, the *bcr1*Δ/Δ *wor2*Δ/Δ mutants have increased biofilm or filamentation capacity, depending on the strain, and increased expression of biofilm-related genes. Those properties suggest that Wor2 and Bcr1 function together as negative regulators of biofilm formation. Our findings argue that Bcr1 can act as both a positive and negative regulator of downstream effector genes in the biofilm network and establish a new connection between the biofilm and white-opaque regulatory networks.

## Introduction

Biofilm formation is central to the survival of *Candida albicans* as a commensal; surface-associated communities are found on mucosae and skin (Noble et al. 2017; Proctor et al. 2023). It is also central to the pathogenicity of *C. albicans*; this organism is a significant cause of device-associated infection (Ramage et al. 2025). Biofilm formation responds to numerous environmental and cellular signals, and its formation and stability require a large set of genes (Nobile and Johnson 2015). Expression of biofilm-promoting genes is coordinated by a network of signaling pathways and transcription factors, referred to as the biofilm regulatory network (Nobile and Johnson 2015; Noble et al. 2017). Many of the biofilm-promoting genes are required for filamentation—the production of elongated hyphae and pseudohyphae—which is vital for biofilm formation in almost every context (Nobile and Johnson 2015; Noble et al. 2017). Because of this connection, many biofilm regulators are also filamentation regulators.

C. *albicans* has 2 major cell type transitions (Noble et al. 2017). One is the switch between yeast-form cells and filamentous cells that is critical for biofilm formation. The second is the switch between white cells and opaque cells (Soll 2024). White cells are conventional ovoid yeast-form cells (called “white” for historical reasons). Opaque cells are ellipsoidal and are required for mating. White and opaque cells differ in numerous ways, a reflection of the many genes that are differentially expressed between the 2 cell types (Soll 2024). Expression of those white-opaque-related genes is coordinated by a network of signaling pathways and transcription factors (Noble et al. 2017; Soll 2024): the white-opaque regulatory network.

The yeast-filament and white-opaque transitions generally occur under different conditions (Noble et al. 2017). For example, filamentation is favored at high temperature, whereas opaque cell formation is favored at low temperature. Mutually exclusive regulatory relationships contribute to this difference: a master regulator of filamentation, Efg1, represses expression of a master regulator of opaque cell formation, Wor1, and vice versa (Noble et al. 2017). It has appeared that regulatory circuits evolved to block one transition when the other takes place.

Recent studies have revised our view of the relationship between biofilm regulators and white-opaque regulators. First, a study of gut commensalism showed that white-opaque regulators Wor2, Wor3, and Wor4 inhibit commensalism, as does filamentation regulator Efg1, whereas Wor1 promotes commensalism (Witchley et al. 2019). Second, Efg1 and Wor1 can form a heterotypic phase-separated condensate in vitro and in vivo (Frazer et al. 2020; Ganser et al. 2023), a surprising interaction if they simply repress one another's expression. Third, in a clinical isolate background that naturally expresses lower levels of *WOR1* RNA than the reference strain SC5314, increased *WOR1* expression promotes biofilm formation and expression of several genes associated with filamentation (Do et al. 2022). This function depends upon the Wor1 amino acid residues that mediate Wor1-Efg1 complex formation. Finally, the white-opaque regulator Wor3 was found recently to function as a positive regulator of biofilm formation and filamentation (Cravener et al. 2023). Therefore, while the biofilm and white-opaque regulatory networks antagonize each other under some circumstances, they act in concert under others. This view makes good sense because many biofilm regulators are known to be required for “sexual” or “unconventional” biofilms formed at low temperature by cells with mating potential (Lin et al. 2013; Srikantha et al. 2013; Soll 2024).

Here, we examine whether the white-opaque regulator Wor2 has a role in biofilm formation. It has 3 previously known roles. First, Wor2 is required for white cells to switch to opaque cells under many growth conditions (Zordan et al. 2007). Its function in this context is to activate the expression of *WOR1*. Second, it promotes gut commensalism, which is probably independent of *WOR1* activation because Wor1 inhibits gut commensalism (Witchley et al. 2019). Third, it is a negative regulator of 2 filamentation-related processes: invasion of agar and production of fuzzy colonies at 42 °C (Homann et al. 2009; Vandeputte et al. 2011). These filamentation-related phenotypes occur at temperatures restrictive for opaque cell production, and are observed in *MTL-*heterozygous cells, which under most conditions cannot produce opaque cells (Soll 2024). The latter observations suggest that there may be a connection between Wor2 and filamentation.

The transcription factor Bcr1 is a second gene product in our study. Bcr1 is among the most extensively characterized biofilm regulators (Nobile and Johnson 2015). It has 2 known roles. First, Bcr1 is required under many conditions for biofilm formation (Nobile et al. 2006; Huang et al. 2019). Its major function is to activate cell surface adhesin genes that include *ALS1*, *ALS3,* and *HWP1* (Nobile et al. 2006). In most biofilm studies, Bcr1 is not required for filamentation. Second, Bcr1 can act as a negative regulator of filamentation (Guan et al. 2013). Its major function is to repress several filamentation activator genes that include *TEC1* and *BRG1*. This role has been reported only in opaque cells. The latter observation suggests that Bcr1 and Wor2 may both be active in the same cells.

Our findings here indicate that Wor2 and Bcr1 function together in the control of filamentation and biofilm formation. The immediate significance of the relationship is 2-fold. First, it argues that Bcr1 can act as both a positive and negative regulator of downstream effector genes in the biofilm regulatory network. Second, it establishes a new connection that weaves together the biofilm and white-opaque regulatory networks.

## Materials and methods

### Strains and culture conditions

Clinical isolates were obtained from BEI resources, NIAID, NIH. *Candida albicans* strains SC5314, P76067, P57055, P87, and P75010 were used in this study (Supplementary Table 1). The strains were stored long-term in 15% glycerol solution at −80 °C. Strains were grown out on YPD solid medium (2% peptone, 2% dextrose, 2% agar, 1% yeast extract) at 30 °C for 48 h before all experiments. The strains were cultured for 18 h in YPD liquid medium (2% peptone, 2% dextrose, 1% yeast extract) at 30 °C in a rotator drum.

### Transformations

Transformations of *Candida albicans* were performed in accordance with the transient CRISPR-Cas9 protocol (Min et al. 2016). All primers and plasmids (Noble and Johnson 2005; Vyas et al. 2015; Min et al. 2016) used in this study can be found in Supplementary Table 1.

#### Deletion of *WOR2*

To delete *WOR2*, the *his1*Δ/Δ or wild type strains of each background listed above were transformed. 1 μg Cas9 DNA cassette, 1 μg *WOR2* sgRNA DNA cassette, 3 μg of *wor2:r1HIS1r* or *wor2:r3NATr3,* and 1 μg of NAT1-5 sgRNA DNA cassette (for the *his1*Δ/Δ strains). Plasmid pV1093 and primers “CaCas9/F” and “CaCas9/R” were used to amplify Cas9. The single guides were amplified using split-joint PCR with pV1093 and round 1 primers “WOR2 sgRNA-_/F”, “sgRNA/R”, “WOR2 SNR52-_/R”, “SNR52/F”, and round 2 using round 1 products, and round 3 utilizes “sgRNA/N” and “SNR52/N” to amplify the full single guide cassette. The *wor2:r1HIS1r1* was generated in amplifying pSN52 or *wor2:r3NATr3* pNAT plasmid with “*WOR2* pSN del/F” and “*WOR2* pSN del/R”. For strains constructed with the pSN52 HIS plasmid, we can use the NAT1-5 sgRNA DNA cassette. This makes it possible to recycle the *NAT1* marker at the *his1Δ*::r3NAT1r3 locus due to Cas9-mediated double-stranded break to the repeat flanked region (Huang and Mitchell 2017). Using this technique, recombination is possible between the direct repeats of the marker, rendering the strain nourseothricin sensitive and leaving only a single copy of the repeat at the recycled locus. Transformants were selected on CSM medium without histidine for pSN52 deletions, and were replica plated onto a YPD plate + 400 mg/mL of nourseothricin (clonNAT, Gold Biotechnology) 48 h later to check nourseothricin sensitivity. Transformants were selected on a YPD plate + 400 mg/mL of nourseothricin (clonNAT, Gold Biotechnology) for pNAT deletions. These colonies were then streaked out for singles, gDNA isolated, and then further genotyped by PCR using primers: “WOR2 chk up/F”, “WOR2 chk int/R,” and either “Cd HIS1 Check int/R” or “NAT1 Check/R”. To confirm double mutant construction for the *wor2*Δ/Δ*bcr1*Δ/Δ the *BCR1* ORF was also screened to confirm genotype using “BCR1 chk int/F” and “BCR1 chk ex/R” (Huang et al. 2019).

#### Deletion of *UME6*

To delete *UME6*, SC5314, or the *wor2*Δ/Δ background was used. 1 μg Cas9 DNA cassette, 1 μg *UME6* sgRNA DNA cassette, and 3 μg of *ume6:r3NATr3*. Plasmid pV1093 and primers “CaCas9/F” and “CaCas9/R” were used to amplify Cas9. The single guides were amplified using split-joint PCR with pV1093 and round 1 primers “UME6 sgRNA/F”, “sgRNA/R”, “UME6 SNR52/R”, “SNR52/F”, and round 2 using round 1 products, and round 3 utilizes “sgRNA/N” and “SNR52/N” to amplify the full single guide cassette. The *UME6:r3NATr3* was generated by amplifying the pNAT plasmid with “*UME6* pSN del/F” and “*UME6* pSN del/R”. Transformants were selected on a YPD plate + 400 mg/mL of nourseothricin (clonNAT, Gold Biotechnology). These colonies were then streaked out for singles, gDNA isolated, and then further genotyped by PCR using primers: “UME6 check up/F”, “UME6 check int/R,” and “NAT1 Check/R”. To confirm double mutant construction for the *wor2*Δ/Δ*ume6*Δ/Δ the *WOR2* ORF was also screened to confirm genotype using “WOR2 chk up/F” and “WOR2 chk int/R”.

### Biofilm assays

Strains were grown in 5 mL of liquid YPD rotating at 30 °C for 18 h. 100 μL of liquid medium YPD was prewarmed to 37 °C in a 96-well plate (Greiner 96 wells Cat#655090). Wells were then inoculated to a final OD600 of 0.05 and incubated at 37 °C for 90 min. Wells were then washed with 1X PBS to remove non-adherent cells, and 100 μL of fresh media was added to each well. Plates were then incubated at 37 °C for 24 h at 60 rpm shaking. Supernatant was then removed, and biofilms were washed again with 1X PBS. Biofilms were fixed by adding 100 μL of 4% formaldehyde in 1X PBS and incubated at room temperature for 1 h. Biofilms were washed once more in 1X PBS and then stained overnight using 5.5 mg/mL of calcofluor-white in 1X PBS. The biofilms were washed the next day with 1X PBS and then clarified using a 50% thiodiethanol and 50% 1X PBS for 1 hour. This was followed by a 1-hour incubation with 100% thiodiethanol. Clarified biofilms were then imaged on a Keyence fluorescence microscope using PlanFluor 20X 0.45/8.80-7.50 mm Ph1 objective with 2X digital zoom. Technical replicates (*n* = 3 or *n* = 4) of the biofilms were imaged within the wells, and apical navigation images were taken to ensure even sampling. Each sample has been replicated in at least 3 independent biofilm assays conducted on different days. Side view projections and biofilm volume measurements were measured using the FIJI software program (ImageJ v1.53). Side-view projections of biofilms were observed from Z-stack images taken 1 μm apart. First, Z-stacks were converted to 32-bit, and the background signal was subtracted using the background subtract plugin. The side-view images were obtained by Z- stack vertical slicing and subsequent maximum intensity projection. The side-view images were rescaled based on the objective used for the Keyence-derived images. Brightness was adjusted, and lookup tables were used for yellow coloration. Biofilm volumes were measured by thresholding the image and running an ImageJ macro code as described at https://visikol.com/blog/2018/11/29/blog-post-loading-and-measurement-of-volumes-in-3d-confocal-image-stacks-with-imagej/.

### Filamentation assays

Cells were grown in 5 mL of liquid YPD rotating at 30 °C for 18 h. Pre-warmed 5 mL of YPD was inoculated to an OD600 of 0.5 from the overnight cultures and incubated for 4 h at 37 °C at 60 rpm. Cells were collected via centrifugation (2800 rpm for 5 min) and fixed in 4% formaldehyde in 1X PBS for 15 min. The samples were then washed 1 time in 1X PBS and then stained using calcofluor white and proteinase-K. Cells were imaged on a Keyence fluorescence microscope using PlanFluor 20× 0.45/8.80-7.50 mm Ph1 objective with 2× digital zoom. Images were taken in triplicate and analyzed in Fiji. Cells were measured using the segment tool in FIJI by measuring the filament unit length either from the yeast cell to the filament tip or between septations. Then, the appropriate pixel-to-m ratio for the objective was used to calculate accurate cell length. At least 100 cells were measured per field of view with the use of Z-stacks to circumvent cell overlap problems. At least 200 total cells were quantified per strain.

### RNA extraction and data analysis

Cells were grown in 5 mL of liquid YPD with rotation at 30 °C for 18 h. The next day, cells were inoculated into 25 mL of prewarmed YPD media at an OD600 of 0.2. Cells were grown for 4 h at 225 rpm in a shaking incubator at 37 °C, then harvested by vacuum filtration and frozen at −80 °C until RNA extraction. Three biological replicates were used for RNA-seq experiments.

RNA extraction was performed according to a previously published method (Huang et al. 2019; Do et al. 2022). Cells were disrupted using Zirconia beads (Ambion, Fisher Scientific, Waltham), and extraction was performed using a 25:24:1 phenol:chloroform:isoamyl alcohol method combined with a Qiagen RNeasy Mini Kit (Qiagen, Venlo, Netherlands). RNA-Seq analysis and processing of raw fastq reads were performed by Novogene. Differential expression was assessed using DESeq2 (v 1.40.2) in R using alpha = 0.05.

### Data interpretation

Interpretations and hypotheses were guided by the comprehensive information at the *Candida* Genome Database (Lew-Smith et al. 2025) and binding site analysis through PathoYeastract (Teixeira et al. 2023). Gene Ontology term enrichment was determined by implementing clusterProfiler (v4.8.1) in R by creating a GO term library using FungiDB Candidaalbicans.Eupath.v68) with the R AnnotationForge package (Wu et al. 2021). Genes were defined by having an adjusted *P-*value of less than 0.05 and a fold change on a log_2_ scale of greater than or less than 1. Only GO categories with a *P*-value of less than or equal to 0.05 were considered significant.

## Results and discussion

### Wor2 function in biofilm formation

**Fig. 1.**
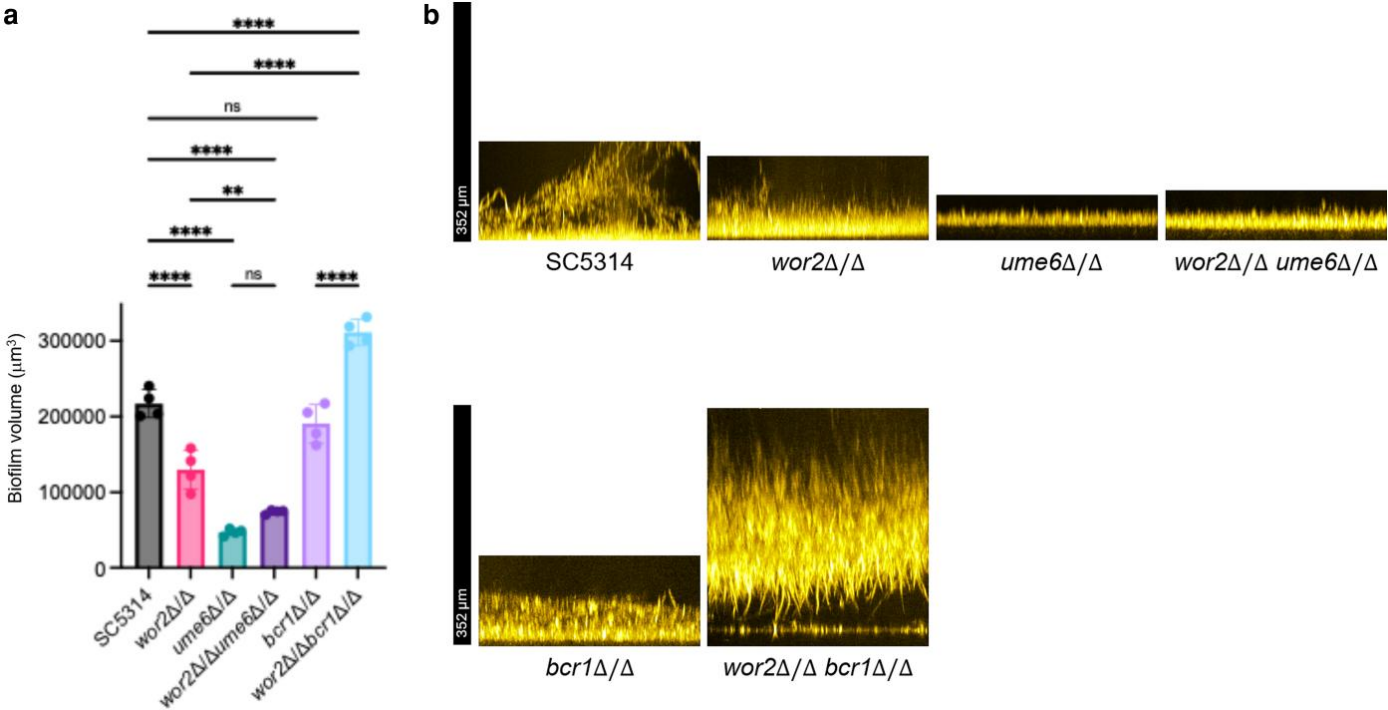
Biofilm assays in SC5314-derived strains. *C. albicans* wild-type, *wor2*  Δ/Δ, *ume6*  Δ/Δ, *wor2*  Δ/Δ  *ume6*  Δ/Δ*, bcr1*  Δ/Δ, and *wor2*  Δ/Δ  *bcr1*  Δ/Δ strains, all in the SC5314 strain background, were assayed for biofilm formation in vitro. Strains were grown in YPD medium in a 96-well plate at 37 °C for 24 h. Biofilms were fixed and stained with calcofluor white and imaged using a Keyence BZ-X800E fluorescence microscope. a) Biofilm volume measurements obtained from 4 independent wells in 96-well plate assays. A one-way ANOVA (Brown-Forsythe test) was used to determine statistical significance, as indicated by asterisks: * = ≤0.05; ** = ≤0.01; *** = ≤0.001; **** = ≤0.0001; ns = not significant. b) Representative biofilm 96-well plate side projection views. The scale bar (352 µm) applies to all images.

Recent studies indicate that white-opaque regulators Wor1 and Wor3 have positive roles in biofilm formation. We hypothesized that other white-opaque regulators may govern biofilm formation as well. To test a potential role for Wor2, we compared biofilm formation for the wild type and a *wor2*Δ/Δ mutant in the genetic background of SC5314 (clade 1, a bloodstream isolate and reference strain). Biofilm production was tested in YPD medium, a moderately inducing condition. Biofilm volume measurements showed that the *wor2*Δ/Δ mutant had a mild but significant defect (Fig. 1a). These results suggest that Wor2 may have a minor role in biofilm formation.

A second gene product may have an overlapping or redundant function with Wor2 (Rutherford 2000; Botstein 2015; van Leeuwen et al. 2017; Laruson et al. 2020; Hunter 2022). In that case, some biofilm formation may occur in a *wor2*Δ/Δ mutant because of the second gene product. Similarly, a mutant lacking the second gene product may have a partially defective phenotype because of the presence of Wor2. In our earlier studies of biofilm regulators, we found that loss of either Bcr1 or Ume6 caused biofilm defects that depend upon strain background or growth conditions. Therefore, we sought to test the redundant function hypothesis with biofilm assays of *bcr1*Δ/Δ *wor2*Δ/Δ and *ume6*Δ/Δ *wor2*Δ/Δ double mutants. A *ume6*Δ/Δ mutant had a significant biofilm defect, and a *ume6*Δ/Δ *wor2*Δ/Δ double mutant behaved similarly (Fig. 1a and b). A *bcr1*Δ/Δ mutant had no significant defect under these conditions, but a *bcr1*Δ/Δ *wor2*Δ/Δ double mutant produced extravagant biofilms with significantly greater volume than biofilms of the wild type (Fig. 1a and b). This outcome was, in essence, the opposite of our expectation if Bcr1 and Wor2 have redundant positive roles in biofilm formation. The results suggest that Bcr1 and Wor2 are redundant negative regulators of biofilm formation, because elimination of both Bcr1 and Wor2 augments biofilm formation.

**Fig. 2.**
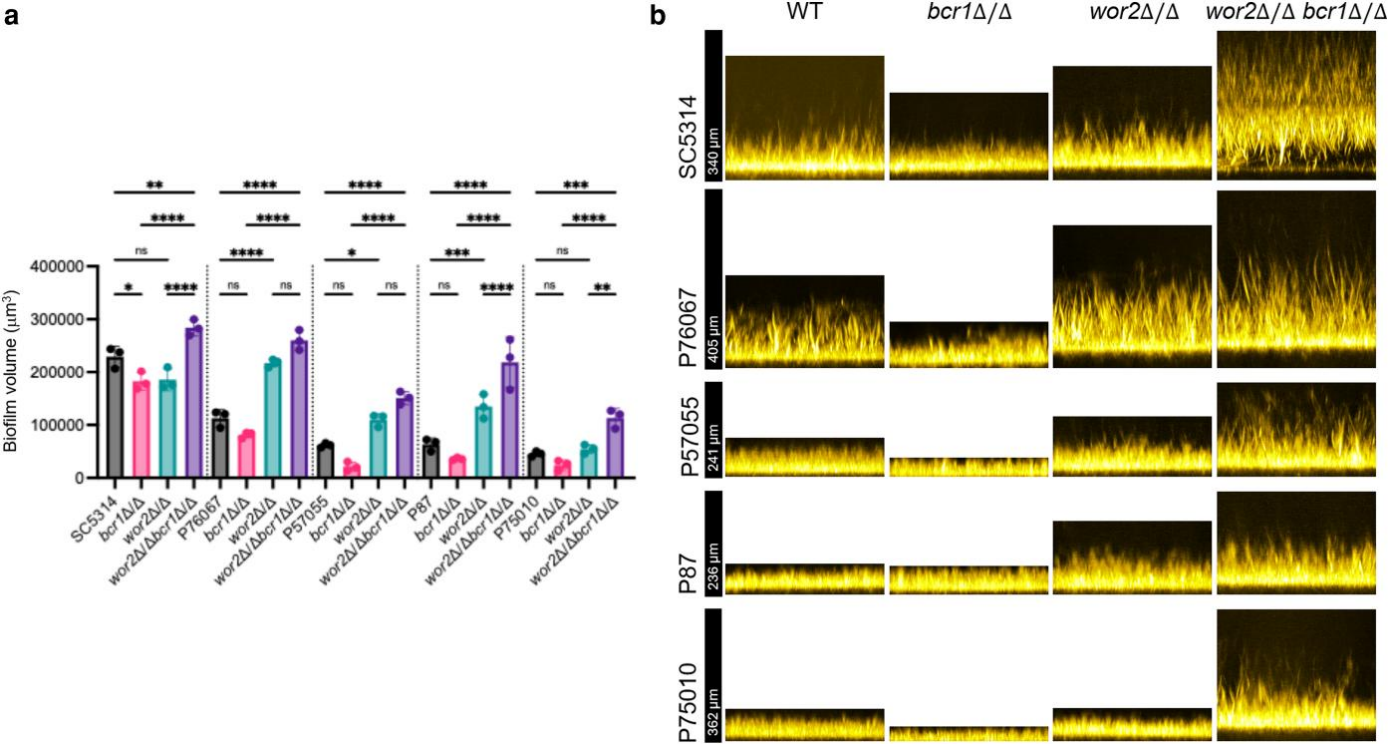
Biofilm assays in multiple strain backgrounds. *C. albicans* wild type, *bcr1*  Δ/Δ, *wor2*  Δ/Δ, and *bcr1*  Δ/Δ  *wor2*  Δ/Δ strains from the SC5314, P76067, P57055, P87, and P75010 backgrounds were assayed for biofilm formation in vitro. Strains were grown in YPD medium in a 96-well plate at 37 °C for 24 h. Biofilms were fixed and stained with calcofluor white and imaged using a Keyence BZ-X800E fluorescence microscope. a) Biofilm volume measurements obtained from 3 independent wells in 96-well plate assays. A one-way ANOVA (Brown-Forsythe test) was used to determine statistical significance, as indicated by asterisks: * = ≤0.05; ** = ≤0.01; *** = ≤0.001; **** = ≤0.0001; ns = not significant. b) Representative biofilm 96-well plate side projection views. The scale bar on the left of each row applies to all images in that row.

We tested the generality of these results with 4 other strain backgrounds: P76067 (clade 2, bloodstream isolate), P57055 (clade 3, bloodstream isolate), P87 (clade 4, oral isolate), and P75010 (clade 11, bloodstream isolate). Biofilm production was compared for each wild type and its respective *bcr1*Δ/Δ, w*or2*Δ/Δ, and *bcr1*Δ/Δ *wor2*Δ/Δ mutants (Fig. 2a and b). The SC5314 strains were included as a control. The *bcr1*Δ/Δ mutants presented little if any biofilm defect under these conditions. The *wor2*Δ/Δ mutants presented increased biofilm formation in the P76067, P57055, and P87 backgrounds, and had no significant defect in the P75010 background. In this set of assays, the SC5314 *wor2*Δ/Δ mutant had no significant biofilm defect, though it trended toward a defect. In all backgrounds, the *bcr1*Δ/Δ *wor2*Δ/Δ double mutant produced more biofilm than the wild type and *bcr1*Δ/Δ single mutants. In the P87 and P75010 backgrounds, the *bcr1*Δ/Δ *wor2*Δ/Δ double mutant produced more biofilm than the *wor2*Δ/Δ single mutant, as it did in the SC5314 background (Fig. 1a and b). There are clearly some strain-dependent effects on phenotype, as we have seen previously (Huang et al. 2019; Do et al. 2022; Cravener et al. 2023; Mao et al. 2023; Sharma and Mitchell 2023; Xiong et al. 2024a; Kim et al. 2024b; Kim and Mitchell 2025). However, all strains present a genetic interaction between *bcr1*Δ/Δ and *wor2*Δ/Δ mutations. In some strains, the *wor2*Δ/Δ mutation suppresses the *bcr1*Δ/Δ mutation. In other strains, the *bcr1*Δ/Δ and *wor2*Δ/Δ mutations together cause a novel phenotype of augmented biofilm formation. These interactions are different, though both are described by the term “epistasis,” as discussed by Roth et al. (Roth et al. 2009).

**Fig. 3.**
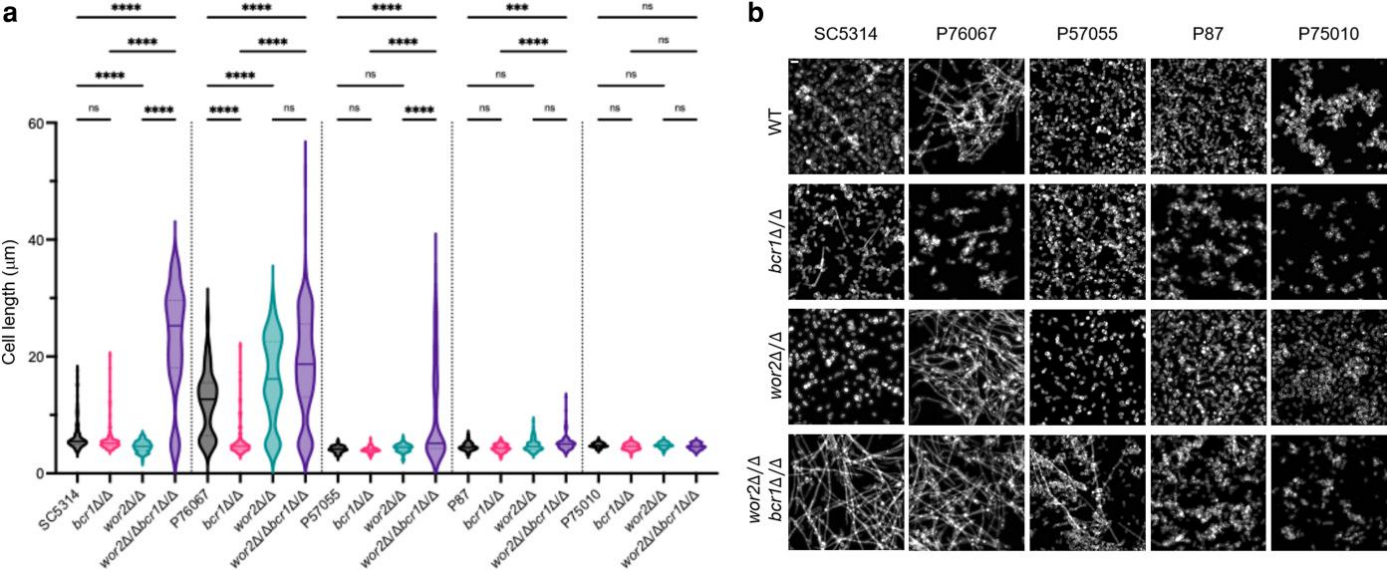
Filamentation assays. Strains were grown in prewarmed YPD medium for 4 h at 37 °C, treated with proteinase K, and stained with calcofluor-white. a) Cell length measurements. Cell length was measured for the strains indicated in at least 3 fields of view, and 100 cells or all cells were measured in each field. A one-way ANOVA (Brown-Forsythe test) was used to determine statistical significance, as indicated by asterisks: * = ≤0.05; ** = ≤0.01; *** = ≤0.001; **** = ≤0.0001; ns = not significant. b) Representative fields of view. The scale bar (in the SC5314 WT image) is 10 µm.

Biofilm formation depends upon production of filamentous cells, including hyphae and pseudohyphae. To see whether Wor2 or Bcr1 influences filamentation, we examined cell samples cultured in YPD for 4 h at 37 °C. In wild-type SC5314, P57055, P87, and P75010, these growth conditions induced little filamentation; in wild-type P76067, there was considerable filamentation (Fig. 3a and b). A *bcr1*Δ/Δ mutation did not affect filamentation in SC5314 and caused reduced filamentation in P76067. A *wor2*Δ/Δ mutation caused increased filamentation in P76067, and not in the other strains. A *bcr1*Δ/Δ *wor2*Δ/Δ double mutation caused increased filamentation compared to wild type in all backgrounds except the minimally filamentous P75010. The *bcr1*Δ/Δ *wor2*Δ/Δ filamentation levels were significantly higher than *wor2*Δ/Δ levels in SC5314 and P57055, and trended higher in P76067 and P87. Clearly, there were strong background effects on the genotype-phenotype relationship. A simple summary is that 1 or both of the genotypes *wor2*Δ/Δ and *bcr1*Δ/Δ *wor2*Δ/Δ can cause increased filamentation in some strain backgrounds.

### Gene expression impact of Wor2 and Bcr1

To explore the basis for the functional interaction between Wor2 and Bcr1, we conducted RNA-sequencing (RNA-seq) analysis. We examined 4 strains, all from the SC5314 background: the wild-type strain and the *bcr1*Δ/Δ, *wor2*Δ/Δ, and *bcr1*Δ/Δ *wor2*Δ/Δ mutants. We used the growth conditions, 4 h in YPD at 37 °C, in which hyperfilamentation of the double mutant was observed.

Comparison of RNA levels for the *bcr1*Δ/Δ *wor2*Δ/Δ double mutant vs the wild type (Supplementary Table 2) indicated that 148 genes were significantly upregulated (Log_2_ fold-change >1, adjusted *P*-value < 0.05), with significant enrichment for GO terms related to interspecies interaction. The categories included genes associated with biofilm formation and filamentation, including hypha-associated adhesin gene *HWP1*. In the same comparison, we found that 70 genes were significantly downregulated (Log_2_ fold-change <−1, adjusted *P*-value < 0.05), with enrichment for GO terms related to biofilm formation. Affected genes included the hypha-associated adhesin gene *ALS3*. It was unexpected that *ALS3* and *HWP1* would be affected oppositely by the *bcr1*Δ/Δ *wor2*Δ/Δ genotype: *ALS3* and *HWP1* are core filamentation genes, which are coregulated under diverse filamentation-inducing conditions (Martin et al. 2013; Azadmanesh et al. 2017).

**Fig. 4.**
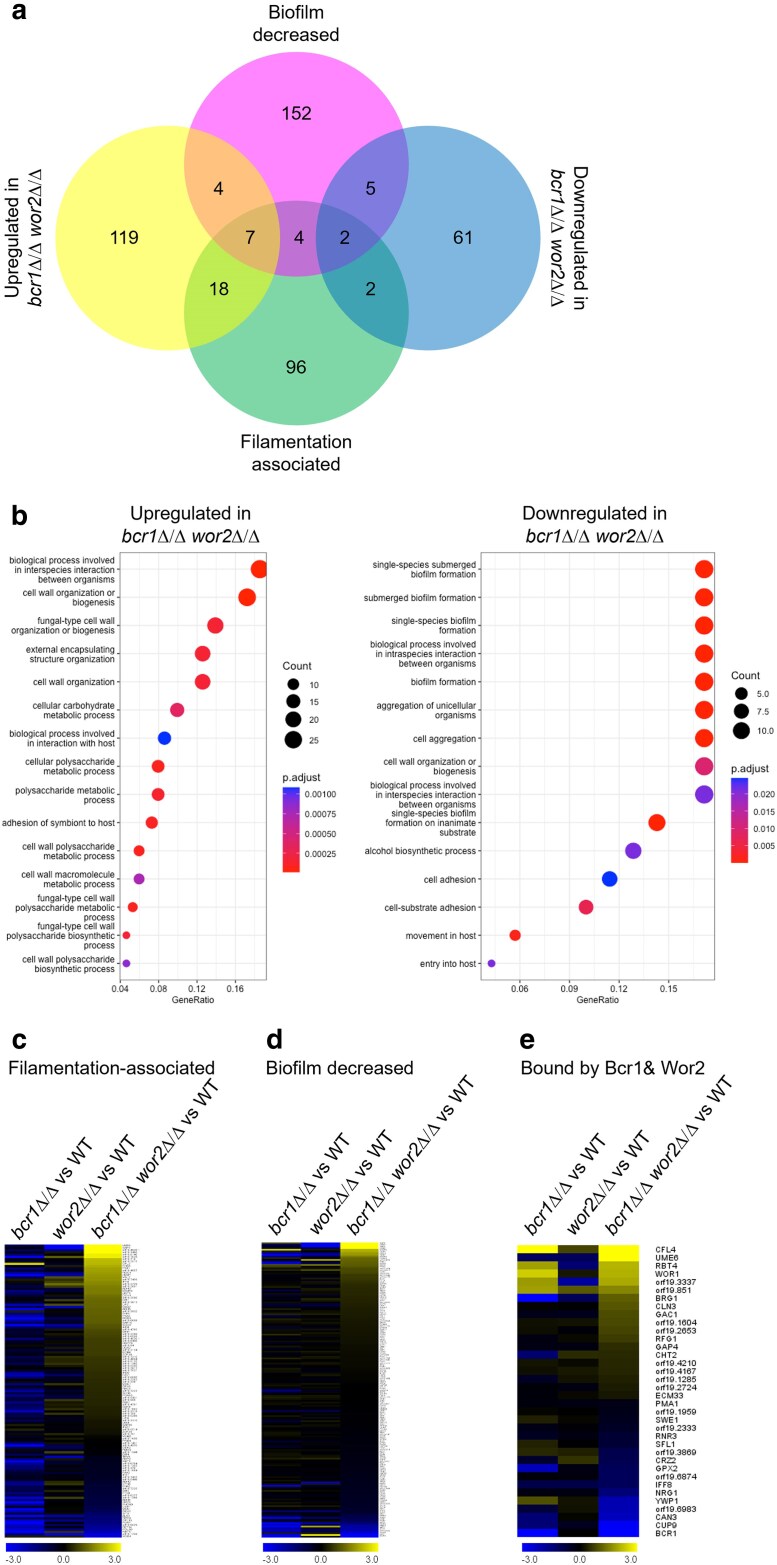
Gene expression changes, based on RNA-sequencing (RNA-seq) analysis. Four strains from the SC5314 background—the wild-type strain and the *bcr1*Δ/Δ, *wor2*Δ/Δ, and *bcr1*Δ/Δ *wor2*Δ/Δ mutants—were grown in prewarmed YPD medium for 4 h at 37 °C. Three independent cultures of each strain were used to prepare RNA, and RNA-seq analysis was performed. The entire dataset is in Supplementary Table 2. a) Gene set overlap. A Venn diagram presents the number of genes upregulated or downregulated in the *bcr1*Δ/Δ *wor2*Δ/Δ mutant vs wild type (Log_2_ fold-change >1 or <−1, adjusted *P*-value < 0.05), and their overlap with filamentation-associated genes (Azadmanesh et al. 2017) and with genes with the “biofilm formation: decreased” descriptor in the *Candida* genome database (Lew-Smith et al. 2025). b) Gene Ontology term enrichment. GO term enrichment analysis using clusterProfiler (Wu et al. 2021) for genes upregulated or downregulated in the *bcr1*Δ/Δ *wor2*Δ/Δ background with a Log_2_ fold-change >1 or <−1 and adjusted *P*-value < 0.05. The dot size represents the number of genes in each category, and categories with a *P* < 0.05 were considered significant. c to e) Heatmaps present Log_2_ fold-changes for RNAs from gene subsets in every single mutant and the double mutant vs wild type. Scales range from a Log_2_ fold-change of −3 (blue) to +3 (yellow), as indicated in the scale bars. c) Impact of *bcr1*Δ/Δ and *wor2*Δ/Δ mutations on filamentation-associated genes. d) Impact of *bcr1*Δ/Δ and *wor2*Δ/Δ mutations on Biofilm formation: decreased genes. e) Impact of *bcr1*Δ/Δ and *wor2*Δ/Δ mutations on genes bound by Bcr1 and Wor2 (Nobile et al. 2012; Do et al. 2025). Only genes that are significantly regulated (Log_2_ fold-change >1 or <−1, adjusted *P*-value < 0.05) in the *bcr1*Δ/Δ *wor2*Δ/Δ double mutant are shown.

To gain functional insight into the impact of the *bcr1*Δ/Δ *wor2*Δ/Δ genotype, we compared RNA levels among the strains for 2 gene subsets (Fig. 4a; Supplementary Table 2): the 129 genes whose expression correlates with filamentation under diverse growth conditions (Azadmanesh et al. 2017), and the 174 genes with the phenotype descriptor “biofilm formation: decreased” in the *Candida* Genome Database (Lew-Smith et al. 2025). Those 2 subsets share only 13 genes (Fig. 4a), so together they represent a broad view of genes related to biofilm formation. Among filamentation-associated genes, only a small fraction had significantly upregulated expression (25/129 with Log_2_ fold-change >1 and adjusted *P*-value < 0.05) in the *bcr1*Δ/Δ *wor2*Δ/Δ strain compared to the wild type (Fig. 4a and b, and Supplementary Table 2). This observation suggests that the hyphae produced by the *bcr1*Δ/Δ *wor2*Δ/Δ strain are not entirely typical of strain SC5314. Among “biofilm formation: decreased” genes, only 11/174 had significantly upregulated expression (Fig. 4a and c, and Supplementary Table 2). The 11 upregulated genes included biofilm regulatory genes *BRG1, HGC1, WOR1, WOR3,* and *UME6*. Each one of these genes can promote filamentation, biofilm formation, or both when overexpressed. Therefore, increased expression of these potent regulatory genes provides a simple explanation for the *bcr1*Δ/Δ *wor2*Δ/Δ mutant phenotype.

### Hypothesis for the combined impact of Bcr1 and Wor2

**Fig. 5.**
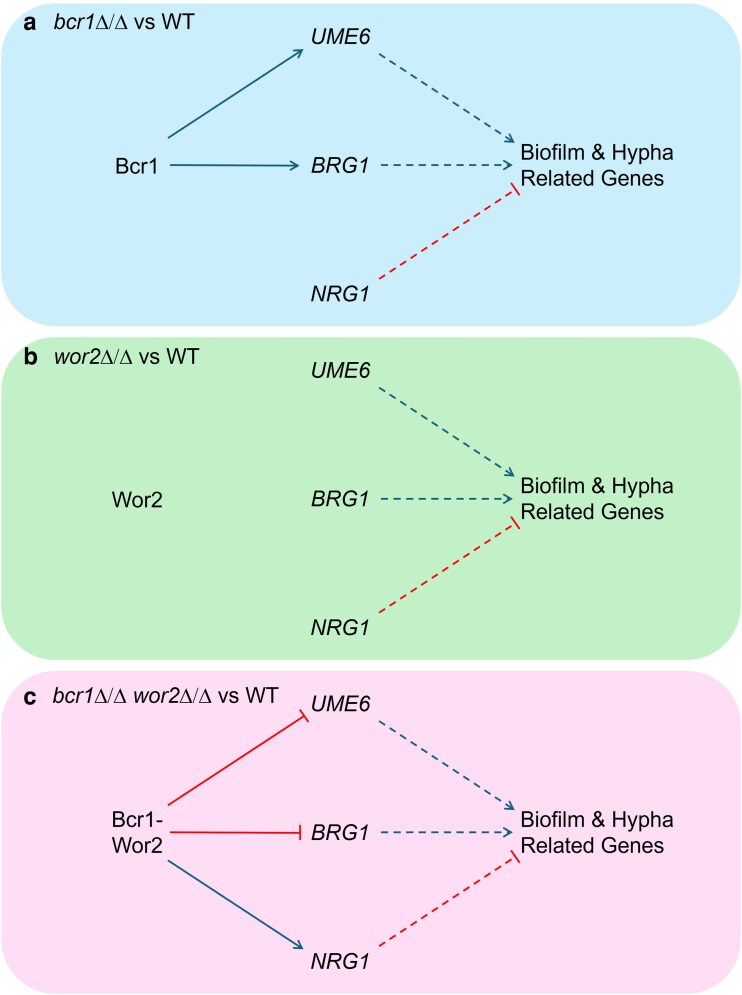
Bcr1, Wor2, and expression of biofilm- and hypha-related genes. Summary diagrams represent findings from (a) *bcr1*Δ/Δ vs wild type, (b) *wor2*Δ/Δ vs wild type, and (c) *bcr1*Δ/Δ *wor2*Δ/Δ vs wild type RNA-seq comparisons. Full dataset comparisons may be found in Supplementary Table 2; gene subset comparisons are summarized in Fig. 4c–e and may be found in Supplementary Table 2. a) In the *bcr1*Δ/Δ mutant, biofilm/hyphal activator genes *BRG1* and *UME6* are significantly downregulated, as are several biofilm- and hypha-related genes. The activator genes *BRG1* and *UME6* are bound by Bcr1 (Nobile et al. 2012), suggestive of direct activation by Bcr1. Expression of biofilm/hyphal repressor gene *NRG1* is not significantly affected in the *bcr1*Δ/Δ mutant. b) In the *wor2*Δ/Δ mutant, biofilm/hyphal activator genes *BRG1* and *UME6* are not significantly altered in expression, nor is biofilm/hyphal repressor gene *NRG1.* c) In the *bcr1*Δ/Δ *wor2*Δ/Δ double mutant, biofilm/hyphal activator genes *BRG1* and *UME6* are significantly upregulated, and biofilm/hyphal repressor gene *NRG1* is significantly downregulated. All 3 of these regulatory genes are bound by Bcr1 (Nobile et al. 2012) and Wor2 (Hernday et al. 2013), suggestive of direct repression (*BRG1* and *UME6*) or activation (*NRG1*) by Bcr1 and Wor2. Several biofilm- and hypha-related genes are upregulated in the *bcr1*Δ/Δ *wor2*Δ/Δ double mutant, but not in either component single mutant, including *DCK1, HGC1, HWP1, HYR1, RBT4, RFX2, SAP5, SAP6,* and *WOR3*. Many of these genes are bound by Brg1 (Nobile et al. 2012) or Ume6 (Do et al. 2025) in chromatin immunoprecipitation experiments or have Nrg1 binding sites from in silico prediction (Teixeira et al. 2023), a suggestion that Brg1, Ume6, or Nrg1 may relay the regulatory signals from Bcr1 and Wor2. Symbols: blue lines with arrowheads reflect positive control (downregulation in the mutant strain); red lines with bars represent negative control (upregulation in the mutant strain); solid lines represent relationships reported in the present study; dashed lines represent relationships observed in previous studies (Murad et al. 2001; Saville et al. 2003; Argimon et al. 2007; Carlisle et al. 2009; Nobile et al. 2012; Noble et al. 2017; Do et al. 2025). A more comprehensive summary of previously known regulatory relationships may be found in Basso et al. (Basso et al. 2019).

What is the molecular basis for target gene regulation by Bcr1 and Wor2? Fig. 5 summarizes the major observations we report here. Our hypothesis is that the key elements are (i) negative control of *UME6* and *BRG1* by Bcr1 and Wor2; (ii) activation of Ume6 and Brg1 effector genes; (iii) positive control of *NRG1* by Bcr1 and Wor2, and derepression of Nrg1 effector genes. We consider each of these points in turn and then discuss briefly the strain variation seen here.

Negative control by Bcr1 and Wor2. Published data (Nobile et al. 2012; Hernday et al. 2013) indicate that Bcr1 and Wor2 bind to the 5′ regions of 16 genes with significantly altered expression (Log_2_ fold-change >1 or <−1 and adjusted *P*-value < 0.05 [omitting *BCR1* itself]) in the *bcr1*Δ/Δ *wor2*Δ/Δ double mutant. Of those, 8 are upregulated, and 8 are downregulated in the double mutant (Fig. 4d, Supplementary Table 2). This observation confirms that Bcr1 and Wor2 are not solely repressors under our conditions, in agreement with their well-established roles as activators under other growth conditions (Nobile et al. 2012; Hernday et al. 2013). Few of the 16 genes have significantly altered expression in the *wor2*Δ/Δ mutant; many have similar expression changes in the *bcr1*Δ/Δ single mutant and the *bcr1*Δ/Δ *wor2*Δ/Δ double mutant (Fig. 4d, Supplementary Table 2). Three genes are striking exceptions: *UME6*, *BRG1,* and *NRG1. UME6 and BRG1* are significantly downregulated in the *bcr1*Δ/Δ mutant, not significantly affected in the *wor2*Δ/Δ mutant, yet are upregulated in the *bcr1*Δ/Δ *wor2*Δ/Δ double mutant. This observation suggests that *UME6/BRG1* regulation by Bcr1 and Wor2 together has a distinct effect—repression—from regulation by either Bcr1 or Wor2 alone.

Activation of Ume6 and Brg1 effector genes. Gene activation by Ume6 and Brg1 has been well established by published studies (Carlisle et al. 2009; Nobile et al. 2012; Do et al. 2025). We seek to make only 1 point here. We correlated gene expression data in our study and published chromatin binding data for Brg1 and Ume6 (Nobile et al. 2012; Do et al. 2025). Of 148 genes that were upregulated in the *bcr1*Δ/Δ *wor2*Δ/Δ strain compared to the wild type, 89 of the genes are bound by either Ume6, Brg1, or both (Supplementary Table 2). We suggest that derepression of *UME6* and *BRG1* in the *bcr1*Δ/Δ *wor2*Δ/Δ double mutant can explain the majority of the double mutant's gene expression impact.

Role of Nrg1. *NRG1* is the third exceptional Bcr1/Wor2 target. Its expression is unaltered in *bcr1*Δ/Δ or *wor2*Δ/Δ single mutants, but it is significantly downregulated in the *bcr1*Δ/Δ *wor2*Δ/Δ double mutant. (Its downregulation may result from increased *BRG1* expression in the double mutant (Cleary et al. 2012)). Nrg1 is well established as a repressor of filamentation genes (Murad et al. 2001; Saville et al. 2003; Noble et al. 2017), though to our knowledge it has not been used in published genome-wide chromatin binding studies. We used deduced Nrg1 binding sequences CACCCT, CCCCCT, ACCCCT, and MVCCCT (Murad et al. 2001; Argimon et al. 2007) in Pathoyeastract (Teixeira et al. 2023) to predict potential Nrg1-bound genes. Of 148 genes that are upregulated in the *bcr1*Δ/Δ *wor2*Δ/Δ strain compared to the wild type, 121 of the genes have 5′ Nrg1 binding sites. Therefore, repression of *NRG1* in the *bcr1*Δ/Δ *wor2*Δ/Δ double mutant may contribute to the mutant's gene expression impact.

Strain variation in the impact of Bcr1 and Wor2. The augmented biofilm and hyperfilamentation phenotypes caused by the *bcr1*Δ/Δ *wor2*Δ/Δ genotype vary in severity among the clinical isolates we tested. Strain variation in filamentation and biofilm formation has been seen repeatedly for wild-type *C. albicans* isolates (see Li et al. 2003; Hirakawa et al. 2015, for example). Perhaps it is not surprising then that we have seen variation in these traits when testing impact in diverse strains of defined mutations (Huang et al. 2019; Do et al. 2022; Cravener et al. 2023; Mao et al. 2023; Sharma and Mitchell 2023; Kim and Mitchell 2025). Variation in the combined impact of 2 mutations may reflect the product of genotype-phenotype variation for each component mutation. However, 1 genotype-phenotype relationship is uniform among all 5 clinical isolates: the genotype *bcr1*Δ/Δ *wor2*Δ/Δ is always associated with increased biofilm formation compared to the wild-type genotype.

There are 2 well-established explanations for natural variation in filamentation of which we are aware. One explanation is that strain SC5314 carries a hyperactive *ROB1* allele (Glazier et al. 2023) that promotes filamentation. This allele is rare (Glazier et al. 2023), so it can account for the strong responses of SC5314 compared to the other strains, but not for those of P76067. A second explanation is that many strains are trisomic for Chromosome 7, which reduces filamentation because of increased expression of *NRG1* (Kakade et al. 2023), which is on Chromosome 7. Our strains are not trisomic for this chromosome, though (see Supplementary Fig. 8 of Hirakawa et al. 2015), so this explanation does not account for the differences we have observed here.

One observation that may seem puzzling is that some strains in our study presented filamentation defects but were able to produce biofilm, as seen by comparing Figs. 2 and 3. Although both assays were conducted in RPMI medium, the conditions were somewhat different. Filamentation was assayed after 4 h in aerated cultures; biofilm formation was assayed after 24 h in a 96-well plate. Filamentation assays are thus conducted under less nutrient-depleted conditions than biofilm assays; filamentation assays are also conducted in a less hypoxic environment than biofilm assays. Both nutrient sensing (Bastidas et al. 2009) and hypoxia (Henry et al. 2021) influence filamentation and adherence. Therefore, differences in the outcomes of these assays are to be expected and have been seen before (Kim et al. 2024a; Xiong et al. 2024b).

## Conclusions

Three main conclusions are established by our work. First, Bcr1 can function as either a positive or negative regulator of biofilm-promoting genes, depending on the strain's genotype. Many transcription factors can function as activators at some genes and repressors at others (see (Mahendrawada et al. 2025) for example), but it is less common for a transcription factor to have these alternate roles at the same gene. There is a similar situation with *C. albicans* biofilm regulator Efg1 (Do et al. 2022); in that case, modest expression differences among other biofilm regulatory genes determine whether Efg1 has positive or negative effects at target promoters. The second conclusion is that Wor2 is connected to the biofilm regulatory network. Here we extended previous studies by defining both its functional interaction with Bcr1 as well as the biofilm-related genes that Wor2 affects. We described circuitry that can explain our observations. It should be considered speculative, though it is grounded in published literature. We note that *Candidozyma auris WOR2* has just been reported to be a negative regulator of biofilm formation in that organism, and *wor2* defects display strain-dependent effects (Louvet et al. 2026). Perhaps the *WOR2-BCR1* genetic interaction is broadly conserved among *Candida* pathogens. The third conclusion is that strain variation can modify substantially the phenotypic impact of Bcr1 and Wor2. This theme has become a constant among genotype-phenotype studies of *C. albicans* regulatory genes (Anderson and Dietz 2024; Lindemann-Perez and Perez 2024), where it has pointed toward new functions and interactions even among well-studied gene products (Do et al. 2022; Mao et al. 2023; Sharma and Mitchell 2023; Xiong et al. 2024a; Kim and Mitchell 2025).

## Supplementary Material

jkag133_Supplementary_Data

## Data Availability

Strains and plasmids are available upon request. The authors affirm that all data necessary for confirming the conclusions of the article are present within the article, figures, and Supplementary materials. RNA-seq data have been deposited in the NCBI Gene Expression Omnibus with accession number GSE318758. Supplemental material available at G3 online.
